# A randomized clinical trial of topical dexamethasone vs. cyclosporine treatment for oral lichen planus

**DOI:** 10.4317/medoral.25040

**Published:** 2021-09-25

**Authors:** Maria Georgaki, Evangelia Piperi, Vasileios I Theofilou, Efstathios Pettas, Eleana Stoufi, Nikolaos G Nikitakis

**Affiliations:** 1 DDS, MSc, PhD, Scientific Associate. Department of Oral Medicine and Pathology and Hospital Dentistry, School of Dentistry, National and Kapodistrian University of Athens, Greece; 2 DDS, MSc, PhD, Assistant Professor. Department of Oral Medicine and Pathology and Hospital Dentistry, School of Dentistry, National and Kapodistrian University of Athens, Greece; 3 DDS, Resident and PhD student. Department of Oncology and Diagnostic Sciences, School of Dentistry, University of Maryland, Baltimore, USA; 4 DDS, Postgraduate Student. Department of Oral Medicine and Pathology and Hospital Dentistry, School of Dentistry, National and Kapodistrian University of Athens, Greece; 5 MD, DDS, PhD, Professor and Chair. Department of Oral Medicine and Pathology and Hospital Dentistry, School of Dentistry, National and Kapodistrian University of Athens, Greece

## Abstract

**Background:**

Oral lichen planus (OLP) is a common, frequently symptomatic, immune-mediated disease. Various treatments have been used for symptomatic OLP, including corticosteroids and immunosuppressants administered topically or systemically. The aim of this study was to compare the effectiveness of topical dexamethasone vs. topical cyclosporine in treatment of symptomatic OLP.

**Material and Methods:**

Thirty-two patients with biopsy-proven symptomatic OLP were randomly assigned to two therapeutic groups: dexamethasone 2mg/5ml or cyclosporine 100mg/ml, both administered topically in a swish and spit method three times a day for 4 weeks. The patients were followed up for a total of 6 months. Assessed parameters included clinical scoring (according to Thongprasom’s scale, 0-5), pain (VAS scale, 0-10), dysphagia and speech difficulties (none, mild or severe). Possible side effects, including fungal overgrowth, were also recorded.

**Results:**

At the end of the 4-week treatment period, both dexamethasone and cyclosporine showed a statistically significant improvement in clinical scoring (*p*<0.025 and *p*=0.034, respectively), which was better with dexamethasone (*p*=0.001). In addition, both dexamethasone and cyclosporine induced statistical significant improvement in pain and dysphagia (and speech difficulties for dexamethasone), without significant differences between the two groups. Regarding side effects, patients in the dexamethasone group developed candidiasis more frequently compared to cyclosporine (*p*=0.031).

At the end of the 6-month follow-up period, the difference in response between the two groups was not statistically significant. Interestingly, a trend for further improvement compared with the end of the 4-week treatment period was noticed only for patients treated with cyclosporine.

**Conclusions:**

Despite the small number of enrolled patients, topical cyclosporine treatment induces a significant clinical improvement in symptomatic OLP patients, which, compared to topical dexamethasone, appears to be less pronounced during initial administration, but capable to induce further improvement after discontinuation with a satisfactory long-term remission in the absence of significant side effects. This study may contribute to a better understanding of the differences in effectiveness of OLP topical treatments and guide future larger scale clinical trials.

** Key words:**Oral lichen planus, topical treatment, dexamethasone, cyclosporine.

## Introduction

Oral lichen planus (OLP) is a T cell-mediated immunological disease of unknown etiology, possibly a response to an unknown antigen, with a global prevalence of 0.5-1.5% in the general population (1,2). OLP predominantly affects the middle-aged population (50-60 years old) and more frequently women (3). Any oral soft tissue site may be affected with a predilection for the buccal mucosa (up to 80% bilaterally), followed by the tongue (lateral borders, ventral and dorsal surface) and the gingiva (1-3).

OLP has several clinical forms, including reticular, papular, plaque-like (or hypertrophic), atrophic, erosive/ulcerative and bullous (1,3-5). Erosive/ulcerative and atrophic forms are often symptomatic with frequent and unpredicTable exacerbations (1,3,6). The accompanying symptoms are of variable intensity and character, ranging from burning sensation to severe pain, also including eating, swallowing or even speech difficulties (1,3,6). Confirmation of diagnosis is based on histopathologic findings, including a dense lymphohistiocytic infiltrate in a subepithelial band-like distribution, accompanied by exocytosis, hydropic degeneration of basal keratinocytes and presence of apoptotic cells (Civatte bodies); hyperkeratosis, hyperplasia or atrophy of the spinous layer and saw-toothed rete pegs are also seen (5).

The successful management of OLP remains challenging (7-14). A complete cure is not currently a realistic goal because of its recalcitrant nature. Multiple treatment options have been suggested and used with variable success, including topical, intra-lesional or systemic corticosteroids (12,15), topical calcineurin inhibitors (including tacrolimus, pimecrolimus and cyclosporine) (16,17), systemic immunosuppressive medications (such as azathioprine and cyclosporine) (18,19), topical or systemic retinoids (20,21), biological agents (22,23), as well as non-medical interventions, such as surgery, laser treatment and photodynamic therapy (24,25). The choice of treatment depends on the severity of the symptoms, the distribution of lesions in the oral cavity, and the overall health and compliance of the patients.

Topical corticosteroids are generally considered as first line treatment for the management of symptomatic OLP (11-13). On the other hand, cyclosporine has been investigated as an alternative topical agent for the management of OLP (16,26-45). The aim of this study was to compare the effectiveness of topical dexamethasone (2mg/5ml oral solution) and cyclosporine (100 mg/ml oral solution) for the treatment of symptomatic OLP. Secondary outcome measures included the evaluation of side effects and particularly the development of candidiasis.

## Material and Methods

- Participants

Thirty-two patients of Greek descent with histologically confirmed OLP were enrolled in the study, including 23 women and 9 men with a mean age of 58.9 years (age range 33-82).

Inclusion criteria were as follows: (i) clinically and histopathologically confirmed OLP (5), (ii) presence of symptoms, corresponding to erosive/ulcerative or atrophic forms of OLP, (iii) wash-out period (i.e. period without treatment) of at least 2 weeks for topical medications and 2 months for systemic medications, (iv) elimination of candida infection; patients with clinical features of candidiasis at presentation were first treated with antifungal medication, the effectiveness of which was confirmed by negative cytologic smear.

Exclusion criteria were as follows: (i) absence of symptoms, corresponding to reticular, papular or plaque-like forms of OLP; (ii) histopathologic evidence of dysplasia; (iii) possibility of other oral lichenoid lesions, such as oral lichenoid contact (e.g. amalgam-related) lesions or drug reactions, cGVHD in transplanted patients, or history of discoid or systemic lupus erythematosus; (iv) pregnant or breast-feeding women; (v) patients on long-term corticosteroid therapy for other reasons, which could not be discontinued; (vi) systemic diseases, i.e. hypertension and renal diseases (to avoid cyclosporine side effects); (vii) recent history of malignancy.

- Study design

A randomised controlled study was designed. The study protocol was approved by the Ethics Committee of the School of Dentistry, National and Kapodistrian University of Athens, Greece. All patients were informed and signed a consent form.

The study was divided in two time periods: period I consisted of 1-month topical treatment; period II was a 5-month follow-up without therapy.

Patients were randomly divided into two groups. The first group received topical dexamethasone (oral solution 2mg/5ml; Rafarm, Athens, Greece) and the second group topical cyclosporine (oral solution 100mg/ml; Sandimmun Neoral, Novartis-Hellas, Greece). Both drugs were administrated as oral solution for mouthrinses 3 times daily for a period of 4 weeks. Each application was noted in a patient diary.

In order to exclude the possibility of a pre-existing candidiasis, so that its potential development as a side effect of topical treatment could be reliably monitored, an oral cytologic smear for candidiasis was performed for all patients at the beginning of the study. Patients with a positive cytologic smear were first treated with systemic antifungal drugs (Fungustatin 100mg/cap; Pfizer Hellas, Greece, once a day after meal for 14 days) and entered the study, following a repeated negative cytologic smear.

Medications were blindly administered to patients of the two groups, so that the attending clinician was not aware of which group each patient belonged.

- Adverse effects

Safety monitoring for all patients included blood tests (complete blood count with differential, blood glucose levels, serum electrolytes and creatinine levels), as well as blood pressure control. At each subsequent visit, patients were also asked to report any abnormal effects that may possibly have been linked to their treatment.

- Study Phases

The two study phases were as follows:

Phase I: The first group of patients received topical dexamethasone 2mg/5ml and the second group of patients received topical cyclosporine 100mg/ml. Both medications were applied as oral mouthrinses without swallowing three times a day for one month, for 5 minutes each time, using 15 ml of the solution measured with a syringe (so all patients applied the same amount and the same dose every day). The patients were informed not to drink, eat or smoke for 1 hour after application. Meticulous oral hygiene instructions were given to all patients, who were carefully instructed how to use the oral rinses.

Phase II: All patients were monitored for 5 months following the completion of the topical treatment.

- Data collection

The total study duration was six months. The patients were followed up every week for the first month, while receiving the topical treatment. Subsequently, follow-up was conducted every 15 days for the second month and once a month for the subsequent 4 months. Assessed parameters included clinical scoring, pain, dysphagia and speech difficulties.

Clinical score: A clinical score was calculated according to Thongprasom’s scale (grade 0 = no lesion/normal mucosa, grade 1 = mild white striae/no erythematous area, grade 2 = white striae with atrophic area <1 cm2, grade 3 = white striae with atrophic area >1 cm2, grade 4 = white striae with erosive area <1 cm2, grade 5 = white striae with erosive area >1 cm) (45).

Pain: Pain was evaluated using a Visual Analogue Scale (VAS) consisted of a 10-cm horizontal line (0 = no pain, 10 = most severe pain experienced). Patients were requested to mark the scale at each visit.

Dysphagia and speech difficulties: Dysphagia and speech difficulties were recorded as present or absent at every visit.

Possible side effects, including fungal overgrowth (confirmed by cytologic smear), were also recorded at each visit and at the end of the treatment.

- Statistical Analysis

Statistical analysis was performed using a nonparametric statistical test. These methods can be applied to variables that do not come from a normal population (normal distribution) and may additionally be applied even in very small samples. Specifically, Mann-Whitney U test was used to analyze pain and speech difficulties, Wilcoxon W and Kruskal-Wallis were used as statistical tests for clinical scores and compared the clinical responses between the two groups. *P* ≤ 0.05 was set as the cut-off level of statistical significance. IBM Statistics 19 (SPSS/PASW) (Norusis, 211) was used for graphical and statistical analysis; Microsoft Excel for processing graphs.

## Results

- Phase I

Dexamethasone group included 18 patients (14F - 4M) with an age range from 33 to 82 years and a mean age of 61.8 year. Cyclosporine group included 14 patients (9F – 5M), their age ranging from 44 to 81 years with a mean age of 59.6 years (Table 1).

**Table 1:**
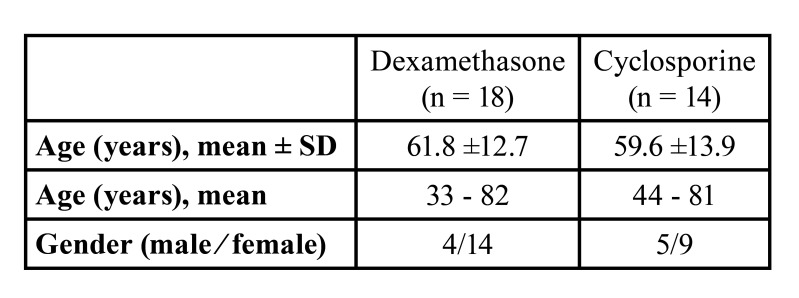
Demographic characteristics of OLP patients per treatment group.

**Figure 1:**
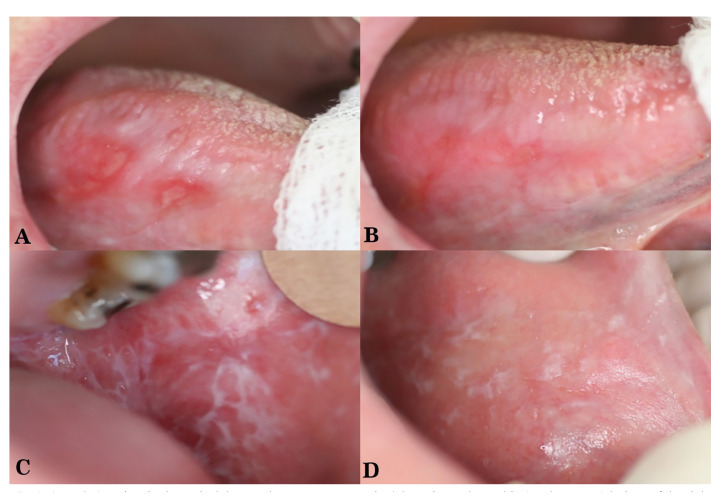
(A and B) Patient in the topical dexamethasone group: Erosive/ulcerative and atrophic (erythematous) lesions of the right lateral border of the tongue (A), showing improvement after one month treatment with topical dexamethasone. (C and D) Patient in the topical cyclosporine group: Atrophic, erosive and reticulated lesions of the left buccal mucosa (C), demonstrating improvement after one month treatment with topical cyclosporine (D).

**Figure 2:**
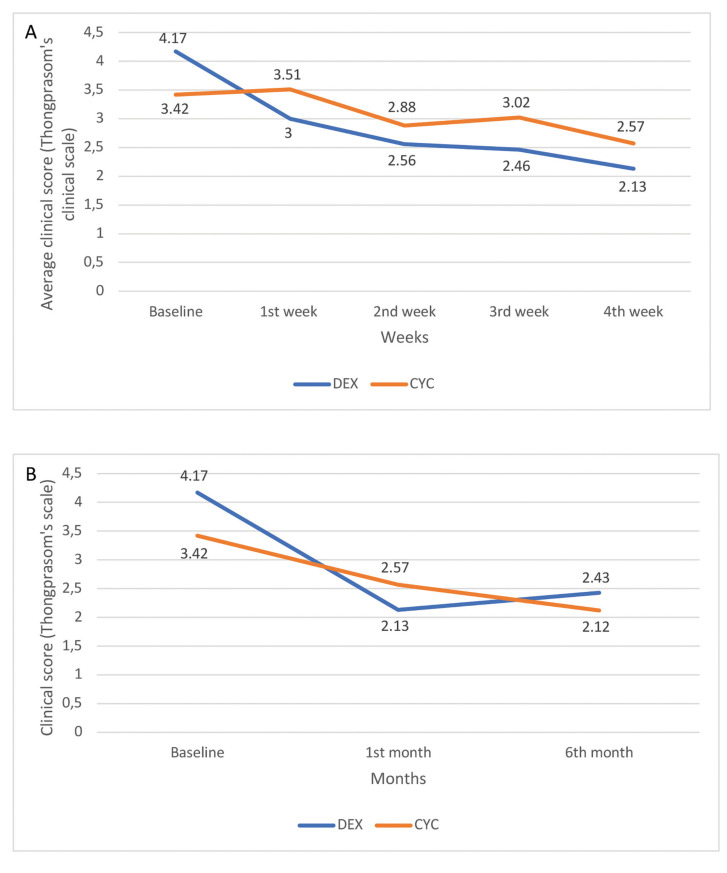
A) Average clinical score according to Thongprasom’s scale at 0, 1, 2, 3 and 4 weeks of treatment for topical dexamethasone vs. topical cyclosporine group; B) Comparison of clinical score according to Thongprasom’s scale at baseline, after 1 month of treatment and after another 5 months of follow-up between topical dexamethasone and topical cyclosporine groups.

Clinical score: The average clinical scores at 0, 1, 2, 3 and 4 weeks of treatment, according to Thongprasom’s scale, were 4.17, 3.00, 2.56, 2.46 and 2.13 for the dexamethasone group, showing significant improvement (*p*<0.025) (Fig. 1, Fig. 2). The corresponding clinical scores at the same time points for the cyclosporine group were 3.42, 3.51, 2.88, 3.02 and 2.57; the improvement was also statistically significant (*p*=0.034) (Fig. 1, Fig. 2). Comparison between the two groups showed that dexamethasone resulted in a better clinical response than cyclosporine (according to Mann-Whitney Test, *p* = 0.001).

**Figure 3:**
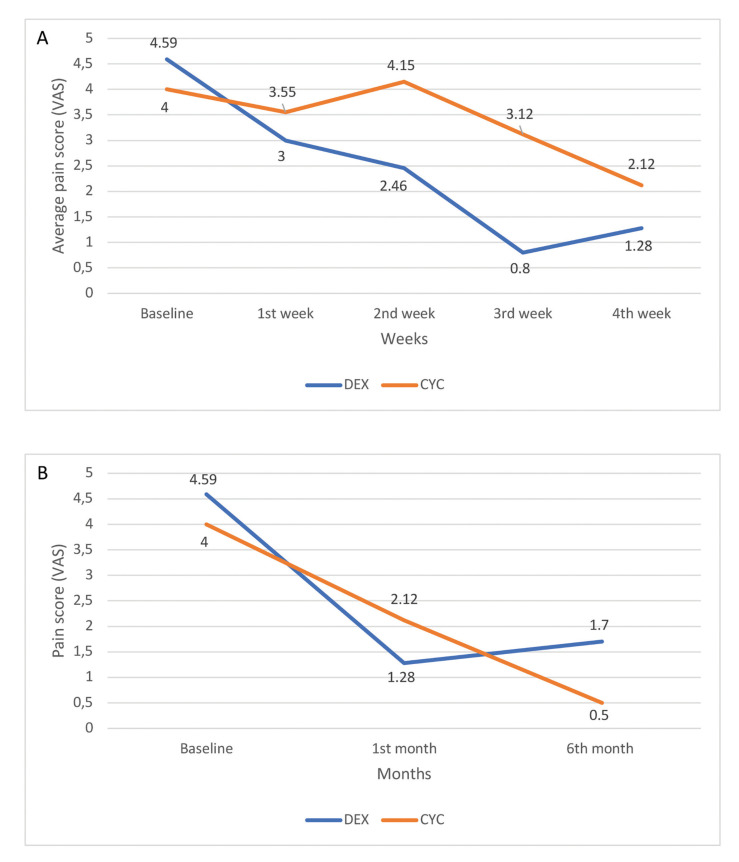
A) Average pain score according to visual analogue scale (VAS) at 0, 1, 2, 3 and 4 weeks of treatment for topical dexamethasone vs. topical cyclosporine group; B) Comparison of pain score according to visual analogue scale (VAS) at baseline, after 1 month of treatment and after another 5 months of follow-up between topical dexamethasone and topical cyclosporine groups.

Pain: At the same time points of treatment as above, the average pain scores according to VAS were 4.59, 3.00, 2.46, 0.80 and 1.28 for the dexamethasone group and 4.00, 3.55, 4.15, 3.12 and 2.12 for the cyclosporine group (Fig. 3). Wilcoxon Signed Ranks Test showed that both dexamethasone (*p*=0.000) and cyclosporine group (*p*=0.018) exhibited improvement in the symptoms of pain. Comparison between the two groups regarding their effectiveness against pain did not show any statistically significant difference (*p*=0.249).

**Figure 4:**
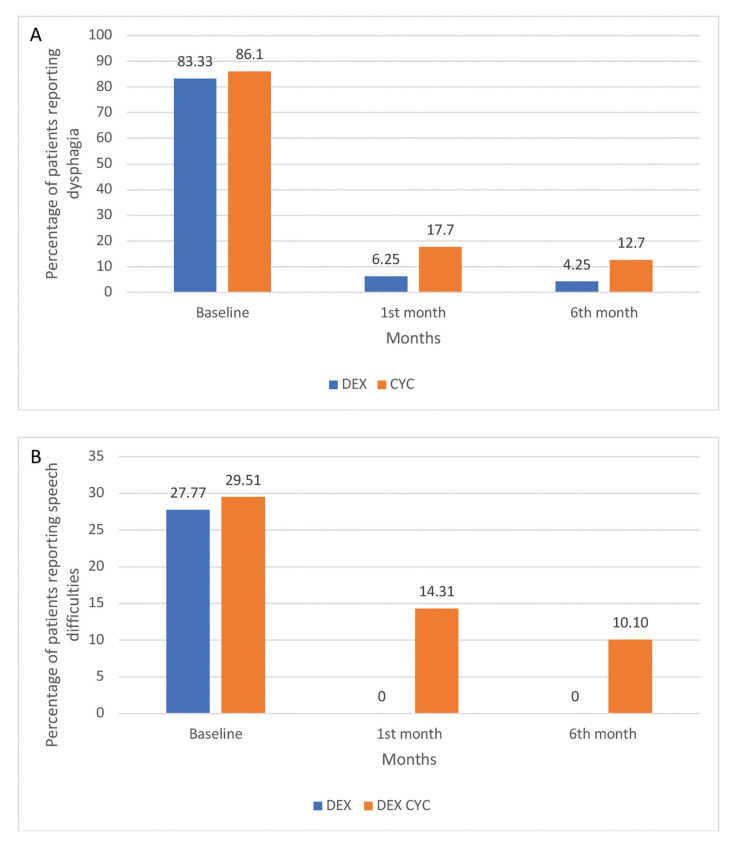
Comparison of percentage of patients reporting (A) dysphagia and (B) speech difficulties, in dexamethasone and cyclosporine group, at baseline, after one month of topical treatment and after another 5 months of follow-up.

Dysphagia and speech difficulties: After four weeks of treatment, the percentage of patients in dexamethasone group reporting dysphagia and speech difficulties was decreased from 83.33% to 6.25% and from 27.77% to 0%; respectively; according to the Wilcoxon Signed Rank Test, these changes were significant (*p*=0.00 and *p*=0.025, respectively). In the cyclosporine group, the corresponding decreases were from 86.10% to 17.70% for dysphagia (*p*=0.026) and from 29.51% to 14.31% for speech difficulties (*p*=0.317) (Fig. 4). The differences between the two groups were not statistically significant (*p*=0.620 for dysphagia and *p*=0.535 for difficulty in speaking).

Side effects: Regarding side effects, the dexamethasone group was associated with candida involvement as 7/18 patients developed candida, in contrast to the cyclosporine group (3/14 patients) (*p*=0.031, according to Mc Nemar test).

- Phase II

Clinical score: At the end of the 5-month follow-up period, the average clinical score, according to Thongprasom’s scale, was 2.43 for the dexamethasone group and 2.12 for the cyclosporine group. According to the Wilcoxon Signed Ranks Test, there was a significant improvement in clinical score for both groups compared to baseline (*p*=0.02 for dexamethasone and *p*=0.017 for cyclosporine). On the other hand, there was not statistically significant difference in clinical scores between the end of the one-month treatment period and the end of the 5-month follow-up period. Also, no statistically significant differences in clinical scores were recorded between groups at the end of the follow-up period (*p* = 0.345) (Fig. 2).

Pain: At the end of the 5-month follow-up period, the average pain score, according to VAS, was 1.70 for the dexamethasone group and 0.50 for the cyclosporine group; for both groups, these scores were significantly lower compared to baseline. No statistically significant differences were found between VAS pain scores recorded at the end of treatment and the end of the follow-up, although a noticeable decrease (from 2.12 to 0.50) was noticed in the cyclosporine group. Also, there was not statistically significant difference between the two groups in pain scores at the end of the follow-up period (*p*=0.052), despite the fact that cyclosporine received a lower average score (Fig. 3).

Dysphagia and speech difficulties: At the end of the 5-month follow-up period, the percentage of patients of dexamethasone group reporting dysphagia and speech difficulties were 4.25% (from 6.25% at the end of treatment) and 0% (also 0% at the end of treatment), respectively. The corresponding percentages of patients reporting dysphagia and speech difficulties in the cyclosporine group were 12.70% (from 17.70% at the end of treatment) and 10.10% (from 14.31% at the end of treatment), respectively; for both groups, these scores were significantly lower compared to baseline. On the other hand, the differences between the end of the one-month treatment period and the end of the 5-month follow-up period were not statistical significant, although a trend for further decrease was noticed (especially for the cyclosporine group). At the end of the 5-month follow-up period, no statistically significant difference in dysphagia or speech difficulties were noticed between the two groups (*p*=0.063) (Fig. 4).

## Discussion

The topical use of corticosteroids has been recommended as the mainstay of treatment for symptomatic OLP and various studies have analyzed the effectiveness of various formulations of topical corticosteroids of different potency (12-15,17,24,43-45). Similarly, topical cyclosporine has been evaluated as an alternative to topical corticosteroids in several studies of variable design, ranging from case reports to randomized double-blind comparative clinical trials (16,26-48). Table 2 summarizes the major findings of the relevant studies. However, the results of these studies are, to some extent, contradictory, due to several reasons, such as differences in study design, including treatment regimens (dosage, method and duration of application), methods of assessment and length of follow-up, as well as the relatively small cohorts of enrolled patients.

Only a few published studies have directly compared topical corticosteroid and topical cyclosporine treatment of symptomatic OLP (43-45). Conrotto *et al*. (44) compared the effectiveness of topical cyclosporine vs. clobetasol in the management of atrophic and erosive lichen planus in 40 patients. Clobetasol was found to be more effective than cyclosporine in clinical improvement after two months of therapy; however, the two drugs had comparable effects on symptoms and clobetasol produced more side effects and less sTable results after 2 months of follow-up.

**Table 2:**
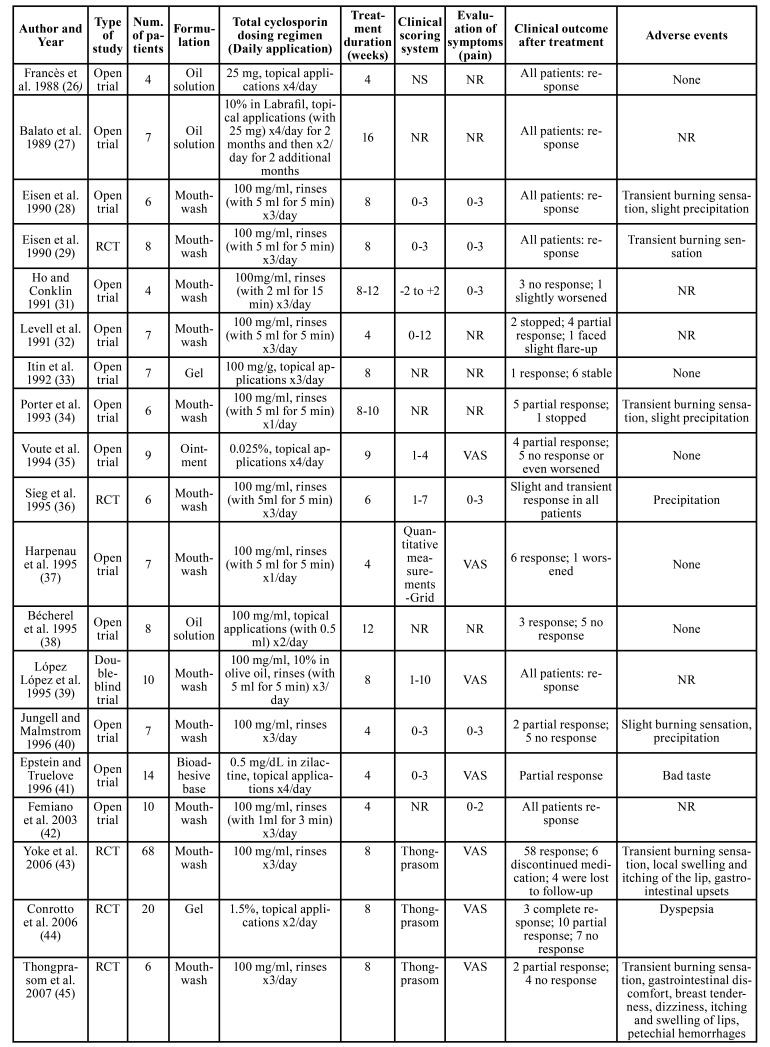
Literature review of all clinical studies using topical cyclosporine for the treatment of oral lichen planus (OLP).

Thongprasom *et al*. (45) compared the effectiveness of cyclosporine solution with triamcinolone acetonide ointment in a small number of Thai patients with symptomatic OLP; no significant differences between the two groups in clinical response or symptoms were recorded. In another study, Yoke *et al*. (43) compared cyclosporine with triamcinolone acetonide for the topical treatment of OLP in a large cohort of 139 patients from Singapore, Korea, India and Thailand, showing that clinical response, pain, burning sensation, area of reticulation, erythema and ulceration at week 4 were better in clobetasol group compared to cyclosporine, but without statistically significant differences between the two groups. Furthermore, the authors pointed that the cyclosporine solution (Sandimmun Neoral) used in most reported trials is an immunosuppressant drug for organ transplant patients and, therefore, it is not specifically formulated and optimized for topical use in oral mucosa, which may compromise its effectiveness.

In most studies, topical cyclosporine was applied as an oral solution (in a swish-and-spit method). In our study, we also assessed the effectiveness of topical cyclosporine in clinical signs (according to Thongprasom’s clinical score scale) and symptoms (pain, dysphagia, speech difficulty), administered as an oral solution and compared with a well-known very potent topical corticosteroid, dexamethasone, also administered as an oral solution in an identical manner, i.e. mouthrinses three times a day for five minutes each time for one month. Interestingly, after one month of treatment, both medications resulted in significant improvement of clinical score; however, the improvement was significantly better in patients receiving dexamethasone. Further, it was noteworthy that patients on dexamethasone showed clinical response even just after one week of treatment with steady further improvement in clinical score during the course of treatment, while patients on cyclosporine did not show any improvement after one week of treatment and, overall, their clinical response rate was slower. As far as symptom reduction goes, both regimens resulted in a significant improvement in pain; although patients using dexamethasone reported a lower average VAS score at all treatment time points compared to those on cyclosporine, the difference between the two groups were not significant. Similarly, the percentage of patients reporting dysphagia was significantly reduced by both medications, while speech difficulties were significantly decreased only in the dexamethasone group. Overall, these findings support a better immediate response of a short-term course of topical dexamethasone compared to topical cyclosporine in the management of symptomatic OLP patients, although cyclosporine is also reasonably effective in controlling clinical signs and symptoms during the one-month treatment period.

Considering that management of OLP aims at controlling symptoms and, ideally, inducing a period of long-term remission, we also evaluated the effects of topical treatment for five months following its one-month course. Noticeably, at the end of the five-months follow-up period, the various parameters (i.e. clinical score, pain, dysphagia and speech difficulties) in both groups did not show significant variations, compared with the corresponding levels at the end of the one-month treatment course; in other words, both topical medications appeared to induce a long-term remission, even without a maintenance regimen. Interestingly, clinical and, especially, pain scores were further reduced for patients of the cyclosporine group during the five-months follow-up period. On the other hand, dexamethasone group patients received relatively higher clinical and pain scores at the end of the follow-up, compared to the corresponding values at the end of the treatment. As a consequence, at the end of the follow-up, the differences in clinical and pain scores between the two groups were not significant (with relatively lower values recorded in the cyclosporine group), both of them showing significant improvement compared to the baseline. Taken together, these results suggest that, in terms of effectiveness, both topical dexamethasone and cyclosporine result in significant improvement in clinical signs and symptoms of OLP, also inducing a long-term remission. However, their profiles and relevant advantages appear to differ: dexamethasone seems to act faster and to induce a more pronounced improvement during its administration with a relatively steady but slowly decreasing long-term effect, while cyclosporine seems to act relatively slower and less effectively during its administration but to result in a better, even improving, long-term remission during follow-up. Interestingly, these observations are in general agreement with those of Conrotto *et al*. (44): in their study, 18 out of 19 patients showed improvement after two months of topical clobetasol treatment, but only 6 out of 18 (33%) were sTable two months after the end of treatment; in contrast, in the group of patients treated with topical cyclosporine, 13 out of 20 showed a clinical response, but 10 out of 13 (77%) were sTable two months after treatment, while another patient with no response during therapy demonstrated late improvement without lesions at the end of the follow-up period. These findings raise the interesting question whether cyclosporine may be more effective compared to topical corticosteroids in inducing long term remission, a valid hypothesis that needs to be further tested in larger studies with even longer follow-up.

Regarding side effects, no major complications were seen in either group in our study. Only development of candidiasis was recorded and was more common in the dexamethasone group, affecting 38.9% of patients, as opposed to 21.4% of patients receiving cyclosporine. This is a well-known side effect of topical immunosuppressive treatment and several investigators suggest the prophylactic use of antifungal medications; for example, Lodi *et al*. (49) have demonstrated the development of candidiasis in one third of OLP patients receiving topical clobetasol treatment for 6 weeks without miconazole prophylaxis and have recommended the use of either antifungal prophylaxis or, especially in patients with negative *Candida* carriage before starting treatment, local anti-infective agents with weak antifungal effect (e.g. chlorhexidine mouthwash). Overall, based on previous investigations and the current study, it can be concluded that the topical application of cyclosporine appears to be safe with little or no toxicity and limited side effects.

With regards to the potential mechanism of cyclosporine function in the management of OLP, it should be considered that OLP is a chronic inflammatory disease with T lymphocytes playing a crucial role in the involved long-lasting inflammatory processes. Further, it is possible that the molecular basis of the disease and the expression of specific molecules differ between the variable clinical forms of OLP. It has been previously shown that high expression of TLR4 and NF-κB p65 in OLP may induce the production of inflammatory cytokines (such as TNF-α and IL-6) and chemokines, which have been shown to be upregulated in OLP (50,51). Ge *et al*. (50) found that steroids (specifically dexamethasone) and cyclosporine can inhibit TLR4 expression, thus negatively regulating NF-κB signaling in an OLP model; in addition, cyclosporine could induce apoptosis and thus inhibit cell proliferation of human keratinocytes. The potential role of NF-κB as a contributor in OLP pathogenesis and as a possible therapeutic target has been also supported by Rhodus *et al*. (52), who noticed a significant decrease in the levels of NF-κB-dependent cytokines (TNF-α, IL-1α, IL-6 and IL-8) in saliva following topical dexamethasone treatment in patients with erosive OLP, as well as a positive correlation between the reduction in IL-1α and IL-8 levels and the improvement in OLP symptoms as assessed by VAS.

Overall and despite the aforementioned differences between studies, topical cyclosporine appears to show comparable effectiveness with topical corticosteroids in the management of symptomatic OLP with limited side effects. It has been pinpointed that the main drawback of cyclosporine is its high cost, even in low concentrations. On the other hand, the use of topical corticosteroids (such as dexamethasone, clobetasol etc.), even in higher dosages and especially for long-term treatment, may be more cost-effective for the patient (44). Therefore, topical steroids can be recommended as the first line therapy for patients with symptomatic OLP because of adequate efficacy, limited side effects and favourable cost-benefit ratio, especially in long-term treatment, although, as concluded by Lodi *et al*. (9) in a systematic review of 28 studies regarding OLP treatment, there may be insufficient evidence to concretely support the superiority of any specific treatment. On the other hand, topical calcineurin inhibitors, including cyclosporine, can be used as a second line therapy. Furthermore, the aforementioned possibility of longer and more sTable remission induced by cyclosporine may counteract or even reverse the cost effectiveness disadvantage; obviously, more comparative studies with longer follow-up, taking into account the cost-benefit ratio in the long run, are in order.

In conclusion, both topical corticosteroids in the form of dexamethasone and topical cyclosporine are effective against symptomatic lesions of OLP. Despite the relatively small number of enrolled patients, topical dexamethasone appeared to correlate with a more pronounced clinical improvement compared to topical cyclosporine during the one-month administration period, while the latter seemed to be associated with a more sTable response during the five-months follow-up period. However, further research and more studies with a larger cohort of patients and controls are needed in order to reach a better understanding on the effectiveness of various topical therapies in symptomatic OLP.
